# Human Coronaviruses: A Review of Virus–Host Interactions

**DOI:** 10.3390/diseases4030026

**Published:** 2016-07-25

**Authors:** Yvonne Xinyi Lim, Yan Ling Ng, James P. Tam, Ding Xiang Liu

**Affiliations:** School of Biological Sciences, Nanyang Technological University, 60 Nanyang Drive, Singapore 637551, Singapore; YVON0016@e.ntu.edu.sg (Y.X.L.); S150004@e.ntu.edu.sg (Y.L.N.); JPTAM@ntu.edu.sg (J.P.T.)

**Keywords:** human coronavirus, virus–host interactions, apoptosis, innate immunity, ER stress, MAPK, NF-κB

## Abstract

Human coronaviruses (HCoVs) are known respiratory pathogens associated with a range of respiratory outcomes. In the past 14 years, the onset of severe acute respiratory syndrome coronavirus (SARS-CoV) and Middle East respiratory syndrome coronavirus (MERS-CoV) have thrust HCoVs into spotlight of the research community due to their high pathogenicity in humans. The study of HCoV-host interactions has contributed extensively to our understanding of HCoV pathogenesis. In this review, we discuss some of the recent findings of host cell factors that might be exploited by HCoVs to facilitate their own replication cycle. We also discuss various cellular processes, such as apoptosis, innate immunity, ER stress response, mitogen-activated protein kinase (MAPK) pathway and nuclear factor kappa B (NF-κB) pathway that may be modulated by HCoVs.

## 1. Introduction

Human coronaviruses (HCoVs) represent a major group of coronaviruses (CoVs) associated with multiple respiratory diseases of varying severity, including common cold, pneumonia and bronchilitis [1]. Today, HCoVs are recognised as one of the most rapidly evolving viruses owing to its high genomic nucleotide substitution rates and recombination [2]. In recent years, evolution of HCoVs has also been expedited by factors such as urbanization and poultry farming. These have permitted the frequent mixing of species and facilitated the crossing of species barrier and genomic recombination of these viruses [3]. To date, six known HCoVs have been identified, namely HCoV-229E, HCoV-NL63, HCoV-OC43, HCoV-HKU1, severe acute respiratory syndrome coronavirus (SARS-CoV) and Middle East respiratory syndrome coronavirus (MERS-CoV); of which, four HCoVs (HCoV-229E, HCoV-NL63, HCoV-OC43 and HCoV-HKU1) are globally circulated in the human population and contribute to approximately one-third of common cold infections in humans [4]. In severe cases, these four HCoVs can cause life-threatening pneumonia and bronchiolitis especially in elderly, children and immunocompromised patients [1,5,6]. Besides respiratory illnesses, they may also cause enteric and neurological diseases [7,8,9,10,11].

SARS-CoV first emerged in 2002–2003 in Guangdong, China as an atypical pneumonia marked by fever, headache and subsequent onset of respiratory symptoms such as cough and pneumonia, which may later develop into life-threatening respiratory failure and acute respiratory distress syndrome [12]. Being highly transmissible among humans, it quickly spread across 29 countries, infecting more than 8000 individuals with a mortality rate of about 10% [13,14]. Originally, palm civets were thought to be the natural reservoir for the virus [15]. However, subsequent phylogenetic studies pointed to the bat origin of SARS-CoV based on sequences of SARS-like virus found in bats [16]. The MERS-CoV epidemic surfaced in Saudi Arabia in 2012 with similar clinical symptoms as SARS-CoV but with a much higher mortality rate of about 35% [17]. Unlike SARS-CoV, which exhibits super-spreader events, transmission of MERS-CoV is geographically limited [12]. In fact, reported cases of MERS-CoV often stem from outbreaks within the Middle Eastern countries or recent travel to the region [18,19].

### Taxonomy, Genomic Structure and Morphology

CoVs are a group of large enveloped RNA viruses under the Coronaviridae family. Together with Artierivirdae and Roniviridae, Coronaviridae is classified under the Nidovirale order [20]. As proposed by the International Committee for Taxonomy of Viruses, CoVs are further categorized into four main genera, *Alpha-*, *Beta-*, *Gamma*- and *Deltacoronaviruses* based on sequence comparisons of entire viral genomes [21,22]. These CoVs can infect a wide variety of hosts, including avian, swine and humans. HCoVs are identified to be either in the *Alpha-* or *Betacoronavirus* genera, including *Alphacoronaviruses*, HCoV-229E and HCoV-NL63, and *Betacoronaviruses*, HCoV-HKU1, SARS-CoV, MERS-CoV and HCoV-OC43 (Table 1).

**Figure 1:**
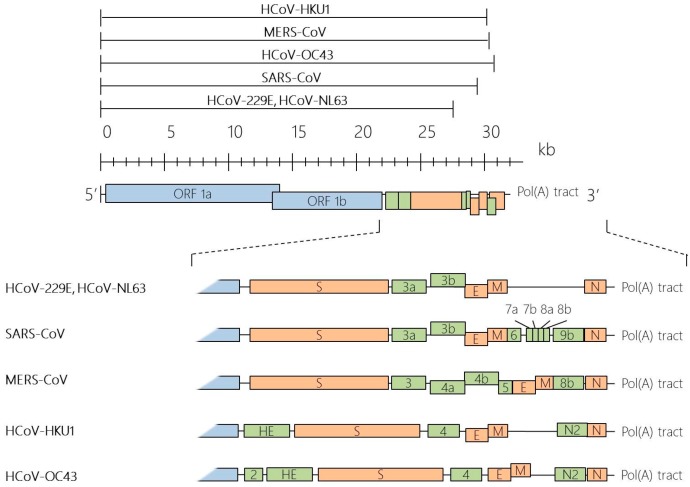
Genome organisation of human coronaviruses (HCoVs). HCoV genomes range from about 26 to 32 kilobases (kb) in size, as indicated by the black lines above the scale. Coronavirus (CoV) genome is typically arranged in the order of 5′-ORF1a-ORF1b-S-E-M-N-3′. The overlapping open reading frames (ORF) ORF1a and ORF1b comprise two-thirds of the coronavirus genome, which encodes for all the viral components required for viral RNA synthesis. The other one-third of the genome at the 3′ end encodes for a set of structural (**orange**) and non-structural proteins (**green**).

Under the electron microscope, the CoV virions appear to be roughly spherical or moderately pleomorphic, with distinct “club-like” projections formed by the spike (S) protein [23,24]. Within the virion interior lies a helically symmetrical nucleocapsid that encloses a single-stranded and positive sense RNA viral genome of an extraordinarily large size of about 26 to 32 kilobases [20]. The positive sense viral genomic RNA acts as a messenger RNA (mRNA), comprising a 5′ terminal cap structure and a 3′ poly A tail. This genomic RNA acts in three capacities during the viral life cycle: (1) as an initial RNA of the infectious cycle; (2) as a template for replication and transcription; and (3) as a substrate for packaging into the progeny virus. The replicase-transcriptase is the only protein translated from the genome, while the viral products of all downstream open reading frames are derived from subgenomic mRNAs. In all CoVs, the replicase gene makes up approximately 5′ two-thirds of the genome and is comprised of two overlapping open reading frames (ORFs), ORF1a and ORF1b, which encodes 16 non-structural proteins. The final one-third of the CoV genomic RNA encodes CoV canonical set of four structural protein genes, in the order of spike (S), envelope (E), membrane (M) and nucleocapsid (N). In addition, several accessory ORFs are also interspersed along the structural protein genes and the number and location varies among CoV species [25] (Figure 1).

## 2. Involvement of Host Factors in Viral Replication and Pathogenesis

**Figure 2:**
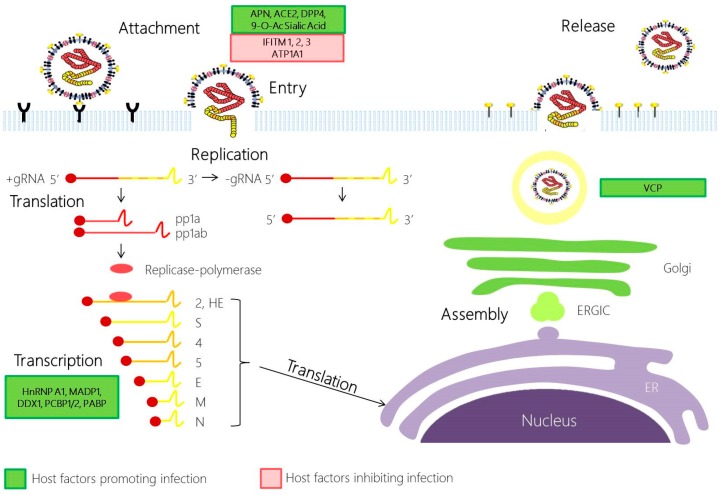
Coronavirus replication cycle. Coronavirus infection begins with the attachment of the S1 domain of the spike protein (S) with its cognate receptor. This drives the conformational change in the S2 subunit in S, promoting the fusion of the viral and cell plasma membrane. Following the release of the nucleocapsid to the cytoplasm, the viral gRNA is translated through ribosomal frameshifting to produce polyproteins pp1a and pp1ab. pp1a and pp1ab are autoproteolytically processed by host and viral proteases to generate 16 non-structural proteins (NSPs), which will then be assembled to form the replicase-polymerase. The replicase-polymerase is involved in the coronaviral replication, a process in which the genomic RNA are replicated and the subgenomic RNA will be transcribed and translated to form the structural proteins. The viral products produced will be assembled in the ERGIC, and bud out as a smooth-wall vesicle to the plasma membrane to egress via exocytosis. Host factors that promote infection and inhibit infection are highlighted in green and red, respectively. APN, aminopeptidase N; ACE2, Angiotensin converting enzyme 2; DPP4, dipeptidyl peptidase 4; 9-*O*-Ac Sialic Acid, 9-*O*-Acetylated Sialic Acid; IFITM, Interferon induced transmembrane protein; ATP1A1, ATPase, Na^+^/K^+^ Transporting, Alpha 1 Polypeptide; HnRNP A1, Heterogeneous nuclear ribonucleoprotein A1; MADP1, Zinc Finger CCHC-Type and RNA Binding Motif 1; DDX1, ATP-dependent RNA Helicase; PCBP1/2, Poly r(C) binding protein 1/2; PABP, Poly A binding protein; COPB2, Coatomer protein complex, subunit beta 2 (beta prime); GAPDH, Glyceraldehyde 3-phosphate dehydrogenase; ERGIC, Endoplasmic reticulum Golgi intermediate compartment; ER, endoplasmic reticulum; VCP, Valosin-Containing Protein.

As intracellular obligate parasites, HCoVs exploit the host cell machinery for their own replication and spread. Since virus–host interactions form the basis of diseases, knowledge about their interplay is of great research interest. Here, we describe what is currently known of the cell’s contribution in CoV infection cycle: attachment; entry into the host cell; translation of the replicase-transcriptase; replication of genome and transcription of mRNAs; and assembly and budding of newly packaged virions (Figure 2). 

### 2.1. Coronavirus Attachment and Entry

CoV infection is initiated by the attachment to specific host cellular receptors via the spike (S) protein. The host receptor is a major determinant of pathogenicity, tissue tropism and host range of the virus. The S protein comprises of two domains: S1 and S2. The interaction between the S1 domain and its cognate receptor triggers a conformational change in the S protein, which then promotes membrane fusion between the viral and cell membrane through the S2 domain. Today, the main host cell receptors utilised by all HCoVs are known: aminopeptidase N by HCoV-229E [26], angiotensin-converting enzyme 2 (ACE2) by SARS-CoV [27] and HCoV-NL63 [28,29], dipeptidyl peptidase 4 (DPP4) by MERS-CoV [30] and 9-*O*-acetylated sialic acid by HCoV-OC43 and HCoV-HKU1 [31,32].

Apart from the conventional endosomal route of entry, some CoVs may also gain entry into the cell via the non-endosomal pathway, or a combination of both. The low pH in the cellular environment and endosomal cysteine protease cathepsins may help to facilitate membrane fusion and endosomal CoV cell entry [33]. Recent evidence has supported the role of cathepsin L in SARS-CoV and MERS-CoV entry [34,35,36]. Other host proteases, such as transmembrane protease serine 2 (TMPRSS2) and airway trypsin-like protease TMPRSS11D, could also perform S1/S2 cleavage to activate the S protein for non-endosomal virus entry at the cell plasma membrane during HCoV-229E and SARS-CoV infection [37,38]. In addition, MERS-CoV is also activated by furin, a serine endopeptidase that has been implicated in the cell entry of other RNA viruses and S1/S2 cleavage during viral egress [39].

Many host cells also utilise its own factors to restrict viral entry. Using cell culture system and pseudotype virus, many groups have identified a family of interferon inducible transmembrane proteins (IFITM), which could inhibit global circulating HCoV-229E and HCoV-NL63 S protein mediated entry, and also the highly pathogenic SARS-CoV and MERS-CoV [12,40]. While the IFITM mode of action remains elusive, cell-to-cell fusion assays performed by some research groups suggest that IFITM3 blocks the enveloped virus entry by preventing fusion of the viral envelope with the plasma membrane or endosomal membranes through modulating the host membrane fluidity [41].

### 2.2. Coronavirus Replication

Following the release and uncoating of viral nucleocapsid to the cytoplasm, CoV replication begins with the translation of ORF 1a and 1b into polyproteins pp1a (4382 amino acids) and pp1ab (7073 amino acids). Here, the downstream ORF1b is translated through ribosomal frameshifting mechanism, in which a translating ribosome shifts one nucleotide in the −1 direction, from the ORF1a reading frame into ORF1b reading frame. This repositioning is enabled by two RNA elements—a 5′-UUUAAAC-3′ heptanucleotide slippery sequence and RNA pseudoknot structure. Subsequently, polyproteins pp1a and pp1ab are cleaved into at least 15 nsp, which assemble and form the replication-transcription complex. With the assembly of the replicase-polymerase, the full-length positive strand of genomic RNA is transcribed to form a full-length negative-strand template for the synthesis of new genomic RNAs and overlapping subgenomic negative-strand templates. These subgenomic mRNAs are then transcribed and translated to produce the structural and accessory proteins. Several heterologous nuclear ribonucleoprotein (hnRNA) family members (hnRNPA1, PTB, SYN-CRYP) have been found to be essential for efficient RNA replication [42]. Other RNA-binding proteins have also been suggested to play a role in CoV replication, such as m-aconitase and poly-A-binding protein (PABP), DDX1, PCBP1/2 and zinc finger CCHC-type and RNA-binding motif 1 (MADP1) [43,44,45].

### 2.3. Coronavirus Assembly and Egress

The assembly of virions is quickly ensued with the accumulation of new genomic RNA and structural components. In this phase of the infection cycle, the helical nucleocapsid containing the genomic RNA interacts with other viral structural proteins (S, E and M proteins) to form the assembled virion. The assembly of CoV particles is completed through budding of the helical nucleocapsid through membranes early in the secretory pathway from the endoplasmic reticulum to the Golgi intermediate compartment (ERGIC). The contributions of the host in this phase of the infection cycle have rarely been explored. Currently, it is known that the M protein orchestrates the entire assembly process by selecting and organizing the viral envelope components at the assembly sites and by mediating the interactions with the nucleocapsid to allow the budding of virions [46]. The M protein interacts with different viral structural proteins, such as the E protein, to assemble into a mature virus. This interaction generates the scaffold of the virion envelope and induces the budding and release of the M protein-modified membrane and with the S protein to assemble the spikes into the viral envelope [46,47]. Following assembly and budding, the virions are transported in vesicles and eventually released by exocytosis. In a recent study, an inhibition of a Valosin-containing protein (VCP/p97) resulted in virus accumulation in early endosome in infectious bronchitis virus (IBV), suggesting a role for VCP in the maturation of virus-loaded endosomes [48].

## 3. Human Coronavirus Infection and Apoptosis

**Figure 3:**
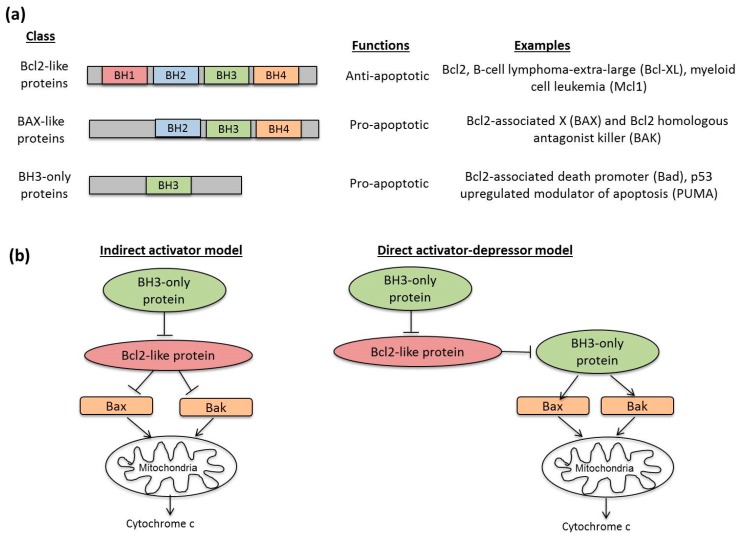
Regulation of MOMP by Bcl2 family of proteins. (**a**) The Bcl2 family of proteins is categorized into three main classes according to their functions and number of Bcl2 homology (BH) domains. The pro-survival Bcl2-like family members (Bcl2, B-cell lymphoma-extra-large (Bcl-XL), myeloid cell leukemia (Mcl1)) contain all four BH domains and are anti-apoptotic. A second class, known as Bcl2-associated X (BAX)-like proteins, which includes BAX and Bcl2 homologous antagonist killer (BAK), is pro-apoptotic and lacks the BH4 domain. Finally, the third class, known as BH3-only proteins (Bid, Bcl2-associated death promoter (Bad), and p53-upregulated modulator of apoptosis (PUMA)), contain only BH3 domain and is pro-apoptotic. (**b**) Two models have been proposed to account for the role of the Bcl2 family proteins in MOMP—the indirect activator model and direct activator–depressor model [11]. In the indirect activator model, the anti-apoptotic Bcl2-like proteins suppress the insertion of Bax-Bak pore complex into the mitochondria to promote MOMP and release of cytochrome c. However, when BH3-only proteins are activated beyond a certain threshold, the inhibitory effects of Bcl2-like proteins can be subverted. In the direct activator–depressor model, BH3-only protein acts as direct activators to induce Bak-Bak insertion into the outer mitochondrial membrane. These BH3-only proteins can be suppressed by Bcl2-like protein, which can in turn be inhibited by another subset of BH3-only proteins. This figure is modified from [12].

Apoptosis is a process of programmed cell death that is tightly regulated and anti-inflammatory. When cells undergo apoptosis, they demonstrate specific hallmarks such as cell shrinkage, extensive plasma membrane blebbing, nuclear pykosis, DNA fragmentation and asymmetrical distribution of plasma membrane [49,50,51]. To date, two main mechanisms of apoptosis have been established—the extrinsic and intrinsic pathways. The extrinsic pathway is initiated by the binding of extracellular death ligands (such as Fas ligand (FasL) and TNF-receptor-related apoptosis-inducing ligands (TRAIL)) to death receptors from the tumour necrosis factor (TNF) super-family [52]. These death receptors then recruit various death adapter proteins, such as Fas-associated death domain protein (FADD) [53], and initiator procaspases 8 and 10 to form the death-inducing signalling complex (DISC) [54,55]. Consequently, the two initiator procapases are cleaved to their active forms and induce a signalling cascade to eventually activate effector caspases 3 and 7. On the other hand, the intrinsic pathway occurs internally in the cell and involves changes in the mitochondrial outer membrane permeability (MOMP) based on the ratio of pro-apoptotic and anti-apoptotic B-cell lymphoma 2 (Bcl2) family of proteins (Figure 3). Enhanced MOMP causes the release of pro-apoptotic factors such as cytochrome c to activate initiator caspase 9. Like the extrinsic pathway, activation of initiator caspase 9 in the intrinsic pathway results in the proteolytic cleavage effector caspases 3 and 7, which in turn process many key cellular proteins essential for apoptosis [56]. Convergence between the two pathways may also occur even before effector caspase activation, when Bid, a pro-apoptotic Bcl2 family protein, is directly cleaved by caspase 8 [57].

During viral infections, apoptosis is induced as one of the host antiviral responses to limit virus replication and production. Many viruses have evolved distinct strategies to subvert apoptosis [58]. For example, some viruses encode for viral proteins that act as Bcl2 family protein homologues [59]. Alternatively, viruses might develop mechanisms to regulate Bcl2 family proteins or caspase activation either directly or indirectly through other molecular pathways such as mitogen-activated protein (MAPK) and nuclear factor kappa B (NF-κB) pathways [60,61,62,63,64,65]. Interestingly, some viruses may engage the apoptotic machineries for efficient viral infection. For instance, alphaviruses and flaviviruses contain phosphatidylserine-rich viral membranes to imitate apoptotic cells to promote viral entry [66].

### 3.1. Cell Tropism and Apoptosis

As HCoVs are respiratory pathogens known to infect tissue cultures and cell lines derived from the respiratory tract, these viruses may also infect other tissue cultures and cell lines. Infection of these tissues and cells may induce apoptosis [67,68]. However, although HCoVs mainly target the respiratory tract during infection, they have also been associated to apoptosis induction in a wide spectrum of cell types, including intestinal mucosal cells, kidney tubular cells and neuronal cells [69,70,71,72,73,74]. Autopsy studies of SARS-CoV-infected tissues revealed apoptosis induced in lung, spleen and thyroid [75]. HCoVs have also been shown to infect the immune system and induce apoptosis in immune cells such as macrophages, monocytes, T lymphocytes and dendritic cells [69,76,77,78,79]. Because these immune cells are associated with the activation of the innate and acquired immunity, it is reasonable to speculate that the massive elimination of these cells could be a viral strategy to suppress the host innate and adaptive immune responses. In a recent study, it was reported that HCoV-229E infection resulted in massive CPE and cell death in dendritic cells [80]. Since dendritic cells are prevalent throughout our bodies, it is possible that they are used as a vehicle to facilitate viral spread and impair our immune systems [80,81].

### 3.2. Molecular Mechanisms in Apoptosis

On a molecular level, HCoV infections have been reported to trigger apoptosis through multiple mechanisms. SARS-CoV-induced apoptosis was shown to be caspase-dependent and could be inhibited by caspase inhibitor Z-VAD-FMK or overexpression of Bcl2 [82,83]. Although viral replication was required for apoptosis induction [83], apoptosis did not affect the viral replication kinetics of SARS-CoV [82]. On the other hand, infection of primary T lymphocytes by MERS-CoV was shown to induce DNA fragmentation and caspase 8 and 9 activation, suggesting that both extrinsic and intrinsic pathways were activated. Unlike SARS-CoV infection, MERS-CoV replication was not necessary to induce apoptosis in infected T lymphocytes [79]. Apoptosis can also be induced by the less pathogenic strains of HCoVs, as substantiated by microarray data showing significant changes in pro-apoptotic and anti-apoptotic gene expression of Bcl2 family members during HCoV-229E infection [84]. Infection of HCoV-OC43 was shown to promote BAX translocation to the mitochondria in human neuronal cells [74]. Although caspases 3 and 9 were activated in HCoV-OC43-infected murine and human neuronal cells [9,74], addition of pan-caspase inhibitor Z-VAD-FMK and the caspase-9 inhibitor Z-LEHD-FMK did not affect the viability of these infected neuronal cells, indicating that programmed cell death induced by HCoV-OC43 could be caspase-independent [74]. This highlights the possibility of a non-classical programmed cell death mechanism induced in HCoV infection.

**Figure 4:**
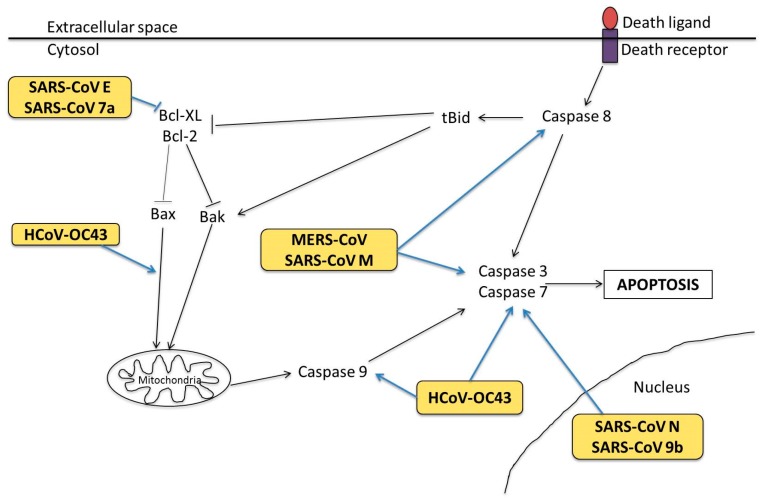
Activation of apoptosis by HCoVs. Binding of death ligands to death receptor induces caspase 8 activation, which in turn activates effector caspases 3 and 7 to stimulate apoptosis. On the other hand, intrinsic pathway is regulated by pro-apoptotic and anti-apoptotic Bcl2 family proteins, such as Bcl-XL, Bcl2, Bax and Bak to induce MOMP. Subsequent caspase 9 activation caused by enhanced MOMP stimulates caspases 3 and 7 activation. During HCoV infection, the virus or specific viral proteins (yellow-orange boxes) target at multiple stages of both the extrinsic and intrinsic apoptosis signalling pathways.

Apoptotic mechanisms during HCoV infection are likely to be manipulated by viral proteins (Figure 4), although this has only mostly been studied in SARS-CoV. Specifically, SARS-CoV S, N, E, M, ORF-6, 7a and 9b proteins have been shown to serve pro-apoptotic functions in their host cells [77,85,86,87,88,89,90,91]. Expression of SARS-CoV E protein and 7a protein promoted mitochondrial-mediated apoptosis by sequestering the anti-apoptotic Bcl-XL protein to the endoplasmic reticulum (ER) membranes [77,92]. SARS-CoV M protein is also highly pro-apoptotic and mediates activation of both caspases 8 and 9 [90]. Additionally, HCoV-OC43 wild type S protein has been shown to induce unfolded protein response (UPR) in human neuronal NT2-N and LA-N-5 cell lines, which may lead to apoptosis [93]. A recombinant HCoV-OC43 harbouring point mutations at its S protein induced stronger caspase 3 activation and nuclear fragmentation than the wild-type virus [93]. It is interesting to note that the localisation of SARS-CoV N and 9b protein is associated with induction of caspase-dependent apoptosis [89,94]. This finding opens up to novel perspectives of the link between subcellular localisation of viral proteins and caspase activation as a mode of apoptosis regulation by HCoVs.

## 4. Human Coronavirus Infection and Innate Immunity

When the cells are exposed to pathogens such as viruses, immune responses are induced as a form of host defence. The immune response is modulated during pathogen exposure in a cell-type dependent fashion. Innate immunity is the first line of defence mounted against the virus before the adaptive immune system is generated. Both the host and virus can manipulate innate immune mechanisms as a form of defence or evasion strategy [95,96].

### 4.1. Pattern Recognition Receptors

Cells in the immune system detect the viral pathogens via several recognition strategies. Of which, the most well characterized is the pattern recognition receptors (PRR), which engage various microbial pathogens via evolutionarily conserved structures known as pathogen-associated molecular patterns (PAMPs). PRRs are mainly categorized into three classes, namely Toll-like receptors (TLRs), retinoic acid-inducible gene I (RIG-I)-like receptors (RLRs) and nucleotide oligomerisation domain (NOD)-like receptors (NLRs).

TLR is a type I transmembrane protein localized to either the cell surface or endosomal vesicles. Their leucine-rich repeats (LRR) domain mediates the recognition of PAMPs and damage-associated molecular patterns (DAMPs) from various sources including bacteria, fungi and viruses [97]. Activation of TLRs occurs mainly in antigen-presenting cells such as dendritic cells (DCs), macrophages, monocytes and B cells. Of the 10 known TLRs in human, TLR2, 3, 4, 7 and 9 are found to be involved in viral detection [98,99]. TLR3 recognizes double-stranded RNA (dsRNA), a replicative intermediate produced during viral RNA replication [100]. TLR7 and 8 detect single-stranded RNA (ssRNA) and TLR9 recognizes unmethylated CpG DNA present in DNA viruses [101,102,103]. Besides nucleic acids, other TLRs, such as TLR2 and 4, sense viral proteins as exemplified in respiratory syncytial virus (RSV), hepatitis virus, measles virus and human immunodeficiency virus [104,105,106,107]. Upon recognition of viral components, TLRs recruit Toll/interleukin-1 receptor (TIR)-containing signalling adaptor molecules, such as MyD88 (myeloid differentiation primary response protein 88) and TIR-domain-containing adapter-inducing interferon-β (TRIF) [108,109,110]. MyD88 and TRIF then stimulate the MAPK and NF-κB pathways to boost IFN and pro-inflammatory cytokine production [111].

**Figure 5:**
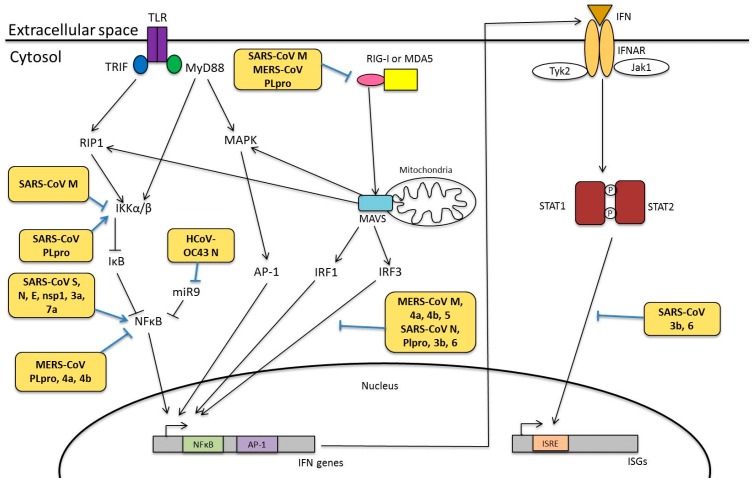
HCoV viral proteins on innate immunity. During HCoV infection, PRRs such as TLRs, RIG-I and MDA5 are activated to trigger a series of signalling pathway, including MAPK and NF-κB, for IFN production. These IFNs then act on IFNAR and activate the JAK-STAT signalling pathway to induce ISGs. The yellow-orange boxes show the viral proteins that have been reported to modulate host innate immunity at multiple stages.

Unlike TLRs, RLRs and NLRs are expressed ubiquitously. RLRs are a family of cytoplasmic receptors that comprise of three members: retinoic acid-inducible gene I (RIG-I), melanoma differentiation associated factor 5 (MDA5), and laboratory of genetics and physiology 2 (LGP2). RIG-I and MDA5 possess two caspase-recruitment domains (CARDs) at their N terminal, a DExD/H-box RNA helicase domain (where x can be any amino acid) and a repressor domain (RD) at the C terminal. On the other hand, LGP2 lacks the CARD domain [112], and it regulates RIG-I and MDA5 either positively or negatively [113,114,115]. RIG-I recognizes 5′-triphosphate moieties present in viral genomic RNA, as well as double-stranded “panhandle” structure formed by self-annealing of complementary ends of the viral genome [116,117]. In contrast, MDA5 usually detects dsRNA sequences of longer length. Binding of RIG-I and MDA5 to the viral RNA causes a conformational change to expose the CARD domain. An adaptor protein known as mitochondrial antiviral signalling adaptor, MAVS, which is localized at the mitochondria and peroxisomes, is recruited. MAVS then activates transcription factors such as interferon regulatory factor 1 (IRF1), IRF3 and NF-κB to trigger the expression of interferons (IFNs) and pro-inflammatory cytokines [117] (Figure 5).

NLRs are another large family of cytosolic proteins that are organized into three main domains: a CARD domain, pyrin domain (PYD), or baculovirus inhibitor repeat domain at the N terminal, a conserved NOD motif at the intermediate region and LRR motifs at the C terminal. The LRR motifs detect viral PAMPs to induce a structural rearrangement. Subsequently, a diverse range of signalling pathways, including MAPK and NF-κB pathways, is activated [118], Furthermore, assembly of multimeric protein complexes known as inflammasomes is mediated by some NLR family members such as NLR family PYD-containing 1 (NLRP1), NLRP3 and NLR family CARD-containing 4 (NLRC4). These inflammasomes activate inflammatory caspase, capase-1, that induces the cleavage of pro-IL-1β and pro-IL-18 into their active forms [119].

### 4.2. Interferon Responses

IFNs are classified into type I, II and III based on their preference for specific IFN receptors. In particular, type I IFN is best known for its antiviral actions. Binding of type I IFNs to the IFN-α/β receptor (IFNAR) induces the oligomerisation of its receptor subunits, IFN-αR1 and IFN-αR2, and consequently conveys downstream signalling via the Janus kinase-Signal Transducer and Activator of Transcription (JAK-STAT) pathway. Autophosphorylation of JAK domains in the IFNAR results in subsequent phosphorylation of STAT1 and STAT2 proteins at their tyrosine residues. This is followed by dimerisation and nuclear translocation of the activated STAT proteins, which recruit IFN regulatory factor 9 (IRF9) to form the IFN-stimulated gene factor 3 (ISGF3). ISFG3 is a transcription factor that binds to its cognate DNA sequence known as IFN-stimulated response elements (ISREs) to activate transcription of IFN-stimulated genes (ISGs) [120] (Figure 5). Many of these ISGs such as 2′–5′ oligoadenylatesynthetase and protein kinase R (PKR) confer resistance against virus invasions [121]. Additionally, type I IFNs facilitates the maturation of dendritic cells (DCs), cytotoxicity of natural killer (NK) cells, and differentiation of T lymphocytes [98].

### 4.3. Modulation of Innate Immunity

Infection by HCoVs, especially the highly pathogenic SARS-CoV and MERS-CoV, is associated with suppression of IFN synthesis [122,123,124,125,126]. The capability of the virus to regulate type I IFN signalling is an important hallmark for virulence [127]. As compared to SARS-CoV and MERS-CoV, a huge rise in type I IFNs were detected in cells infected with HCoV strain 229E [80,124,128].

Based on studies from SARS-CoV and Mouse Hepatitis Virus (MHV)-infected cells, two mechanisms have been proposed to explain the HCoV-mediated inhibition of type I IFN production [13,126]. Firstly, CoV genomic and subgenomic RNA replication takes place in double membrane vesicles to prevent detection by PRRs [13,129]. Secondly, proteins encoded by the virus could interfere with innate immune pathways [13,130]. The structural proteins, nonstructural proteins and accessory proteins of HCoVs have been shown to modify innate immune responses (Figure 5).

#### 4.3.1. Viral Proteins Involved in Innate Immunity

##### Structural Proteins of HCoVs

Expression of SARS-CoV M protein could suppress type I IFN production mediated by RIG-I, but not MDA5, in infected HEK293 cells [131], likely through its first transmembrane domain. However, this inhibition was not observed when expressing the M protein of HCoV-HKU1, suggesting that this activity is not conserved among all HCoV strains [132]. In another study, it was shown that the MERS-CoV M protein could also suppress type I IFN by inhibiting the translocation of IRF3 into the nucleus, although the exact mechanism has not yet been elucidated [133]. Additionally, SARS-CoV N protein was also shown to interfere with the function of IRF3 [134]. The N protein of SARS-CoV likely act at the initial recognition stage of viral RNA via its RNA binding activity, although it neither forms a complex with RIG-I nor MDA5 [135]. This implies that the N protein possibly acts on other viral RNA recognition strategies of the host.

##### Non-Structural and Accessory Proteins of HCoVs

Besides the structural proteins, other nonstructural proteins (nsp) and accessory proteins of HCoVs have also been implicated in the modulation of innate immunity. For instance, nsp1 of both SARS-CoV and MERS-CoV has been demonstrated to modify capped non-viral RNAs to facilitate endonucleolytic cleavage of host messenger RNA (mRNA) [136,137]. Additionally, SARS-CoV nsp1 interacted with the 40S subunit of ribosome to prevent host mRNA translation [136]. This induces the host shutoff mechanism, as transcription and translation of viral RNA are more favoured over that of host mRNA. In a recent study, several residues of SARS-CoV nsp1 were identified to affect IFN-dependent signalling [138]. In addition to nsp1, SARS-CoV and MERS-CoV nsp3 proteins, which possess papain-like protease (PLpro) domain and a PLP2 domain, also antagonize IFN production. Both SARS-CoV and MERS-CoV PLpro domains are deISGylating enzymes and they downregulated mRNA levels of pro-inflammatory cytokines including CCL5, IFNβ, and CXCL10 [139]. Suppression of IFN responses by SARS-CoV PLpro is not mediated by its protease activity. Rather, SARS-CoV PLpro inhibited the phosphorylation of interferon-regulatory factor 3 (IRF3) and its translocation to the nucleus to enhance IFN gene transcription [140]. Expression of MERS-CoV PLpro also antagonizes IFN production and is required for suppression on RIG-I and MDA5 [139,141]. Furthermore, it has been identified that the ADP-ribose-1-monophosphatase macrodomain encoded within nsp3 in HCoV-229E and SARS-CoV is responsible for suppressing IFN induction [142].

Despite being dispensable in viral replication, HCoV accessory proteins are essential in diverse cellular signalling, such as cell proliferation, apoptosis and interferon signalling [25]. In SARS-CoV, ORF3b and -6 are shown to interfere with IFNβ synthesis by inhibiting the phosphorylation and nuclear translocation of IRF3. Furthermore, these accessory proteins also disrupt IFN signalling by preventing IFNβ-induced activation of interferon-stimulated response element (ISRE) found in the promoter region of ISG [134]. The accessory proteins of MERS-CoV, ORF4a, -4b and -5, could similarly suppress IRF3 nuclear translocation, hence significantly reducing IFN-β promoter-driven luciferase activity in cells transfected with these accessory proteins [133].

## 5. Human Coronavirus and ER Stress Response

**Figure 6:**
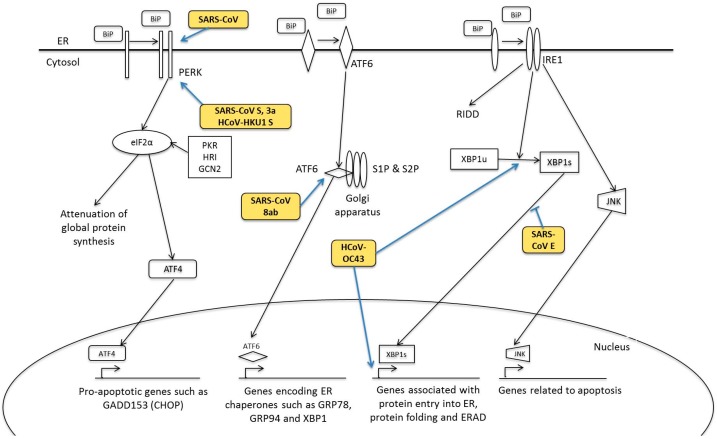
HCoVs on ER stress response. During HCoV infections, the ER stress response, which comprises of three signalling pathways, PERK, ATF6 and IRE1, is activated. HCoVs encode many viral proteins (yellow-orange boxes) that target the various signalling pathway of ER stress during viral infections.

The endoplasmic reticulum (ER) is a cellular organelle important for protein synthesis, folding, processing and post-translational modifications. In normal circumstances, the ER can be loaded with a very high concentration of proteins without perturbing its unique luminal environment [143]. However, when the protein load exceeds the ER folding and processing capacity, rapid accumulation of misfolded or unfolded proteins occurs within the ER lumen. Various signalling pathways, collectively known as ER stress response or UPR, are activated. These pathways are initiated by three ER transmembrane sensors-protein-kinase-R (PKR)-like endoplasmic reticulum kinase (PERK), inositol-requiring protein 1 (IRE1) and activating transcriptional factor 6 (ATF6) to orchestrate the restoration of ER homeostasis by enhancing protein folding, attenuating protein translation and upregulating genes related to protein folding, chaperoning and ER-assisted degradation (ERAD) (Figure 6). In cases of prolonged and irreversible ER stress, apoptosis mechanisms are triggered [144]. During viral infections, ER stress response is induced. This massive utilisation of the ER elicit immense burden, causing the host to mount UPR as its antiviral response [145].

### 5.1. PERK Signalling Pathway

Activation of PERK is initiated by its dissociation of the luminal domain from the ER chaperone, binding immunoglobulin protein (BiP). This is followed by the oligomerisation and autophosphorylation of PERK. In its active form, PERK phosphorylates Ser51 at the α-subunit of eukaryotic initiation factor 2 (eIF2α) to attenuate protein translation [146]. Activation of PERK plays a pro-survival role in cells, as clearly demonstrated by PERK−/− mouse embryonic fibroblasts, which exhibited higher cell death when treated with cycloheximide, an ER stress-inducing agent [147]. Phosphorylated eIF2α not only triggers a shutdown of global protein synthesis, but also enhances the translation of activating transcription factor ATF4 [148]. ATF4 stimulates target gene expression such as GADD153 (also known as CHOP or C/EBP-homologous protein), to enhance transcription of pro-apoptotic genes [149]. Additionally, eIF2α can be phosphorylated by other kinases such as PKR, heme-regulated inhibitor kinase (HRI), and general control non-derepressible-2 (GCN2) [144]. These kinases activate various downstream signalling pathways, which together form the integrated stress response [146,150].

PKR and eIF2α phosphorylation was detected in SARS-CoV-infected cells and inhibition of PKR using antisense peptide-conjugated phosphorodiamidate morpholino oligomers did not affect eIF2α phosphorylation but significantly reduced SARS-CoV-induced apoptosis. SARS-CoV protein replication and virus production were not affected by PKR knockdown. Therefore, it is likely that SARS-CoV adopts a strategy to counteract against the antiviral effects of PKR, thus enabling viral mRNA translation to proceed regardless of eIF2α phosphorylation. PERK was also found to be activated during SARS-CoV infection [75], possibly through its S and 3a proteins [151,152]. In another study, it was demonstrated that expression of a dominant-negative PERK mutant, that inhibited PERK kinase activity, suppressed the transcriptional activation of Grp78 and Grp94 promoters mediated by S proteins of SARS-CoV and HCoV-HKU1 [153]. However, PERK activation is unlikely to occur in all HCoV strains. In neuronal cells lines infected with HCoV-OC43, it was shown that eIF2α was only transiently phosphorylated at the early stage of infection, but was subsequently suppressed and returned back to its basal level of phosphorylation, similar to the mock-infected cells [93]. On the other hand, previous studies from this group showed that PKR, PERK and eIF2α were moderately induced at the early stages of IBV infection, but were subsequently suppressed at late infection stages [154,155]. Nevertheless, the moderate and transient increase in eIF2α phosphorylation was sufficient to activate ATF4 protein translation and upregulate the downstream targets of ATF4, ATF3 and GADD153. Knockdown of PKR and PERK in IBV-infected cells attenuated IBV-induced GADD153 upregulation and IBV-induced apoptosis, although the viral protein replication was unaffected [155]. Upregulation of GADD153 is postulated to induce pro-apoptotic protein TRIB3 and inhibit pro-survival ERK protein [154], as well as provide a negative feedback to rapidly dephosphorylate eIF2α at late stages of IBV infection [155]. Based on these findings, we speculate the HCoVs might use similar mechanism to modulate the PKR/PERK/eIF2α pathway in infected cells. More studies could be done on HCoV infection to analyze the activation of the PKR/PERK/eIF2α pathway at various stages of infection.

### 5.2. ATF6 Signalling Pathway

Like PERK, activation of ATF6 is initiated by dissociation from the ER chaperone, BiP, although alternative mechanisms such as deglycosylation and reduction of disulphide bonds could occur [156,157]. ATF6 then translocates into the Golgi apparatus, where it is proteolyzed by site-1 and site-2 proteases (S1P and S2P). The processed ATF6 then migrates to the nucleus where it turns on expression of genes containing an ER stress response element (ERSE) in their promoters [158]. Like ATF4, ATF6 also induces expression of ER chaperone proteins such as GRP78, GRP94 and transcription factors CHOP and X box-binding protein 1 (XBP1) [150]. XBP1 is essential for IRE signalling [159].

As compared to the two other UPR branches, PERK and IRE1, the ATF6 branch is less well studied. As GRP94/78 are also target genes of ATF6, and their promoter activities were enhanced by SARS-CoV S protein, one could hypothesize that ATF6 pathway could also be induced by SARS-CoV S. Surprisingly, overexpression of SARS-CoV S protein did not affect ATF6 promoter luciferase activity [152]. Deletion of E protein in recombinant SARS-CoV also did not significantly activate ATF6 [91]. Intriguingly, 8ab protein, an accessory protein of SARS-CoV, was shown to reside in the luminal surface of the ER surface and activate ATF6 via facilitating its proteolysis and translocation of the processed ATF6 into the nucleus [160]. 8ab protein from SARS-CoV was found in civet cats and early human isolates, but was subsequently split into two accessory proteins, 8a and 8b, with a characteristic 29-nucleotide deletion [161].

### 5.3. IRE1 Signalling Pathway

IRE1 is believed to be the last UPR branch to be activated in cells undergoing ER stress [162]. It is also the most conserved among all UPR arms [163]. Although IRE1 was initially proposed to be activated in the same mechanism as PERK [162], later studies suggested that the N-terminal luminal domain (NLD) of IRE1 can directly bind unfolded proteins [164,165]. Activation of its RNase domain results in unconventional splicing of a 252-nucleotide intron from homologous to Atf/Creb1 (HAC1) mRNA in yeasts and a 26-nucleotide intron from X-box binding protein 1 (XBP1) mRNA in humans [166]. Splicing of XBP1 generates a potent transcription factor, XBP1s, that induces expression of genes related to protein entry into the ER, folding and ERAD [159]. In a negative feedback mechanism, XBP1s also promotes the transcription of E3 ubiquitin ligase synoviolin to enhance IRE1 ubiquitination [167]. The unspliced variant XBP1u contained a nuclear exclusion signal to sequester XBP1s from the nucleus, thus making XBP1u another negative feedback regulator of XBP1s. [168]. In a separate mechanism, IRE1 can cleave ER-associated mRNA species through regulated IRE1-dependent decay (RIDD) during late stages of ER stress [145]. It is believed that initial XBP1/HAC1 splicing by IRE1 promotes survival but subsequent activation of RIDD upon prolonged ER stress leads to cell death, thus allowing IRE1 to play dual role in apoptosis [169,170]. Another important enzymatic activation of IRE1 is its kinase activity. The kinase domain of phosphorylated IRE1 recruits the TNF receptor-associated factor 2 (TRAF2), which then activates other kinases to eventually activate the c-Jun N-terminal kinase (JNK) and regulates ER stress-dependent apoptosis [171].

Previous studies have investigated the role of IRE1-XBP1 pathway during SARS-CoV infection. Although no increase in XBP1 splicing was observed in SARS-CoV-infected cells [172], deletion of E protein in recombinant SARS-CoV resulted in significant XBP1 splicing and higher rate of apoptosis [91]. On the other hand, infection of HCoV-OC43 caused an induction in XBP1 splicing and enhanced expression of genes regulated by XBP1s, namely Edem, Herp, Grp94 and P58-ipk. However, introduction of two point mutations (H183R and Y241H) in the S protein of HCoV-OC43 led to a higher degree of XBP1 cleavage, followed by a strong activation of caspase-3 and nuclear fragmentation [93]. Since IRE1 pathway is closely associated to JNK activation, it is possible that the JNK pathway is also implicated during HCoV-OC43 infection.

Similar to HCoV infections, it has been shown that the IRE1-XBP1 pathway is activated during IBV infection. Knockdown of IRE1 using specific siRNA in IBV-infected cells augmented IBV-induced apoptosis; however, an opposite effect was observed by XBP1 knockdown in IBV-infected cells. Consistent with the knockdown experiments, transient overexpression of the full-length IRE1α attenuated IBV-induced apoptosis. When both spliced and unspliced forms of XBP1 were overexpressed in IBV-infected cells, the spliced form of XBP1 was shown to be anti-apoptotic and the unspliced form was pro-apoptotic. Overexpression of a dominant-negative XBP1 enhanced IBV-induced apoptosis. Therefore, our findings showed that the anti-apoptotic function of IRE1 during IBV infection could be mediated by its splicing of XBP1, hence converting XBP1 from a pro-apoptotic to anti-apoptotic form. Finally, IRE1 induction during IBV infection was shown to mediate JNK hyperphosphorylation and Akt hypophosphorylation to potentiate the IBV-infected cells to apoptosis [173].

## 6. Human Coronavirus and MAPK Pathways

**Figure 7:**
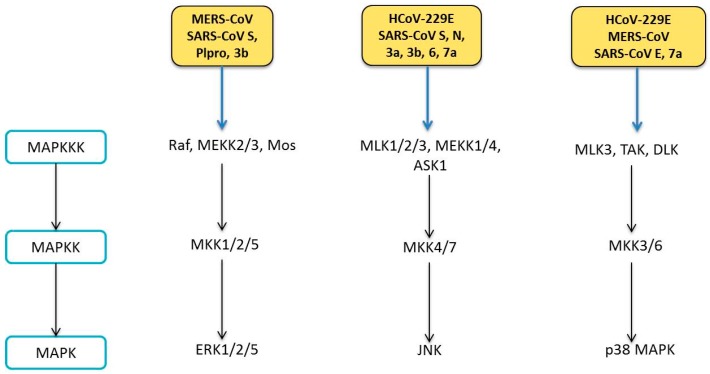
HCoVs on MAPK signalling pathways. MAPK pathways comprises of the ERK, JNK and p38 MAPK pathways. During HCoV infections, the signals are transduced by the MAPK pathways by a three-tier protein kinase cascade, with the kinase of each tier being phosphorylated by upstream kinases at the Thr and Tyr residues. HCoVs and their viral proteins (yellow-orange boxes) have been shown to induce these MAPK pathways as shown in the figure.

The MAPKs are a group of evolutionally conserved serine/theronine kinases that are activated in response to environmental stresses including oxidative stress, DNA damage, cancer development and viral infections [174,175,176]. To date, multiple MAPK pathways have been identified in mammals, and they can be broadly classified into three major categories—the extracellular signal-regulated kinase (ERK), p38 MAPK and stress-activated protein kinase/c-Jun N-terminal kinase (SAPK/JNK) [177]. In all MAPK pathways, the signals are transduced downstream via a three-tier protein kinase cascade. In each tier, the kinases are activated by upstream kinases by dual phosphorylation at the Thr-X-Tyr motif (X represents any amino acid). Presence of extracellular stimuli triggers the activation of MAP kinase kinase kinases (MAPKKKs), which then activate the MAP kinase kinases (MAPKKs). These sequential phosphorylation events eventually activate MAPKs which in turn regulate a variety of fundamental cellular processes such as cell proliferation, survival, motility, differentiation, autophagy, apoptosis and regulation of cytokine production (Figure 7) [178]. 

### 6.1. Modulation of MAPK Pathways

Phosphorylation of all three MAPK members has been detected in cells infected with SARS-CoV [179,180]. Additionally, the MAPK pathways are also activated during infection by other HCoVs, as discussed below (Figure 7).

#### 6.1.1. ERK Pathway

Activation of ERK and its upstream kinases, MEK1/2, was detected in cells infected with SARS-CoV or overexpressed with SARS-CoV S protein [179,181]. However, ERK activation did not contribute to phosphorylation of its downstream target, p90 ribosomal S6 kinase (p90RSK) in SARS-CoV-infected Vero E6 cells [182]. This could be attributed to the higher phosphorylation levels of ERK1 compared to ERK2 in the SARS-CoV-infected cells, as ERK1 has been shown to suppress p90RSK phosphorylation and functional activity [183]. In another recent study, vimentin, a type III intermediate filament protein, was shown to be critical for entry of SARS-CoV, via direct interaction with the viral S protein [184]. Since vimentin could associate with β-adrenergic receptor to regulate ERK activation, one could speculate that induction of ERK pathway by SARS-CoV S protein might be through the interaction between vimentin and the ACE2 receptor, which is required for entry of SARS-CoV [185]. Additionally, binding of SARS-CoV S protein to ACE2 receptor stimulates upregulation of chemokine (C–C motif) ligand 2 (CCL2) mediated by ERK/AP-1 activation. CCL2 is believed to be responsible for respiratory inflammatory symptoms in SARS patients [181]. SARS-CoV S protein-induced ERK phosphorylation was shown to enhance IL8 release [186]. Other viral proteins of SARS-CoV have also been shown to induce ERK activation. Expression of SARS-CoV PLpro was shown to increase ERK1 ubiquitin-mediated degradation to suppress IFN-induced responses [187]. SARS-CoV 3b protein was also involved in ERK phosphorylation to potentiate AP-1 dependent activity of pro-inflammatory cytokine monocyte chemoattractant protein-1 (MCP-1) [188]. Besides SARS-CoV, MERS-CoV infection is also associated with enhanced ERK phosphorylation profile. The use of ER pathway inhibitor inhibited MERS-CoV infection by approximately 50% [189]. Although ERK phosphorylation was not significantly affected by HCoV-229E infection, the level of phosphorylated ERK can be enhanced by the use of chloroquine, a known antiviral agent against viruses [190]. Therefore, targeting the ERK pathway might have significant antiviral potential during HCoV infection.

#### 6.1.2. JNK Pathway

Phosphorylation of JNK and its upstream kinases, MKK4/7, was detected in SARS-CoV-infected cells [191]. Overexpression of SARS-CoV 3a and 7a protein increased JNK activation and augmented IL8 promoter activity [192]. SARS-CoV 3b protein was shown to induce JNK/c-Jun/AP-1 activation to mediate transcription of MCP-1 [188]. Concurrently, the S protein of SARS-CoV was also shown to induce the activation of protein kinase epsilon via JNK activation [193]. Expression of SARS-CoV N protein is associated with the downregulation of prosurvival factors and apoptosis induction in COS-1 cells, possibly mediated by JNK activation [86]. Apoptosis induced by SARS-CoV 6 and 7a protein in Vero E6 and COS-7 cells were blocked by a JNK inhibitor [88]. Therefore, these findings suggest that JNK could act as a pro-apoptotic protein during SARS-CoV infection. However, in another study, phosphorylation of JNK was required for the maintenance of Vero E6 cells persistently infected with SARS-CoV. As persistent infection is only established after apoptotic events, it was proposed that JNK might act as a pro-apoptotic during acute phase of infection, but subsequently switched to become anti-apoptotic during prolonged infection [191]. It is uncertain if these observations noted were dependent on cell-type specificity. In our recent study, our group has found out that JNK was also activated during HCoV-229E infection and serves an anti-apoptotic role via modulation of Bcl2 family proteins. Furthermore, JNK contributes to the production of IFNβ and IL8 in HCoV-229E-infected cells (unpublished). The anti-apoptotic role of JNK during HCoV-229E infection contradicts our observations in H1299 cells infected with animal coronaviruses such as IBV, in which JNK has been shown to be pro-apoptotic [194]. The discrepancy of the involvement of JNK in CoV infections could be attributed to the different virus strains used in the experiments.

#### 6.1.3. p38 MAPK Pathway

Activation of p38 MAPK has been reported in cells infected with SARS-CoV, MERS-CoV and HCoV-229E [180,189,190]. The upstream kinases of p38 MAPK, MKK3/6 were also shown to be phosphorylated [179]. On the other hand, downstream effectors of p38, MAPK-activated protein kinase-2 (MAPKAPK-2), cAMP response element-binding protein (CREB) and activation transcription factor-1 (ATF-1), were also activated in SARS-CoV-infected cells. SARS-CoV-induced phosphorylation of eukaryotic initiation factor 4E (eIF4a) was attenuated by p38 MAPK inhibitor. Although eIF4a is involved in protein translation, SARS-CoV viral protein translation and viral replication was not attenuated by p38 MAPK inhibitor, suggesting that SARS-CoV did not ultilise p38 for viral protein synthesis [180]. Furthermore, SARS-CoV infection induced p90RSK Ser380 phosphorylation, which could be partially negated by p38 MAPK inhibitor. Analysis of individual SARS-CoV proteins demonstrated that transfection of its 7a protein induced p38 activation when fused with GFP [195]. Since SARS-CoV 7a and 3a protein co-immunoprecipitated, they are likely to co-interact in virus-infected cells [196]. Therefore, it is possible that simultaneous co-expression of 7a and 3a proteins might further enhance p38 phosphorylation. In a recent study, the PBZ-binding motif (PBM) of SARS-CoV E protein was demonstrated to bind to syntenin and redistributes it to the cytoplasm, where it acts as a major scaffolding protein for p38 signalling cascade. Inhibition of syntenin using specific siRNA in cells transfected with functional E protein led to decreased p38 MAPK signalling [197]. Consistent with cell culture studies, phosphorylated p38 MAPK level was found to be increased in leukocytes of SARS patients. Enhanced p38, but not ERK, activation likely contributes to elevated IL8 levels and abnormal cytokine profile in SARS patients [198]. Additionally, p38 might also be involved in HCoV replication, as exemplified by HCoV-229E. Inhibition of p38 by its inhibitor SB203580 suppressed the HCoV-229E-induced cytopathic effects and significantly reduced viral titres in a dose dependent manner [190]. The use of chloroquine was also shown to exert antiviral effects against HCoV-229E infection, possibly via its attenuation on p38 activation [190].

## 7. Human Coronavirus and NF-κB Pathway

The NF-κB proteins are a family of transcription factors that regulate expression of genes to control a broad range of biological processes, such as cell death, inflammation, innate and adaptive immune responses. Mammalian NF-κB family composes of five members, RelA (also named p65), RelB, c-Rel, NF-κB1 p50, and NF-κB2 p52, which form dimers in the cytoplasm. It has been established that NF-κB pathway is often targeted by viral pathogens to enhance viral replication, host cell survival and host immune evasion [199,200]. There are two main pathways for NF-κB signalling—the canonical and non-canonical pathways. 

In the canonical pathway, the latent NF-κB forms a complex with its inhibitor IκB protein and is sequestered in the cytoplasm. As mentioned above, presence of viral pathogens activate various membrane sensors such as RIG-I, which induces the phosphorylation IκB by IκB kinase (IKK) complex and its subsequent ubiquitination. IKK complex consists of trimeric subunits including two catalytic subunits, IKKα and IKKβ, and a regulatory subunit, IKKγ (also named NF-κB essential modulator or NEMO). NF-κB is thus released from the inhibitory effects of IκB and translocates to the nucleus, where it stimulates transcription of target genes, either alone or in combination with other transcription factors including AP-1, Ets, and Stat [201]. On the other hand, the non-canonical pathway is independent of IκB degradation, but instead relies on inducible p100 processing. Activation of NF-κB inducing kinase (NIK), a MAPKKK, induces the phosphorylation and activation of IKKα dimeric complex, in turn activating p100. This results in the release of p52/RelB heterodimer from sequestration by p100. The p52/RelB heterodimer translocates to the nucleus to activate target genes related to a number of cellular functions, in particularly cell proliferation, survival and innate immunity [202].

### 7.1. Modulation of NF-κB Pathway

NF-κB pathway has been shown to play an important role in HCoV infections. NF-κB was activated in lungs of mice infected with recombinant SARS-CoV [203]. In the same study, however, subsequent treatment of these infected lung cells with NF-κB inhibitors did not affect virus titres but reduce expression of TNF, CCL2 and CXCL2, hence suggesting that NF-κB is essential for SARS-CoV-mediated induction of pro-inflammatory cytokines [203]. HCoV-229E was also shown to mediate IL8 induction in peripheral blood mononuclear cells (PBMC), which could be attenuated by NF-κB inhibitor [204]. Modulation of NF-κB is mediated via several viral proteins of HCoVs (Figure 5).

#### 7.1.1. Structural Proteins

In SARS-CoV, the S, M, E and N structural proteins have been demonstrated to interfere with NF-κB signalling. A recent study reported an enhanced nuclear NF-κB activity in PBMCs treated with a purified and recombinant SARS-CoV S protein. Synthesis and secretion of IL8 in these S protein-treated cells could be suppressed by NF-κB inhibitor, hence suggesting that NF-κB regulates pro-inflammatory cytokine levels in these cells [204]. SARS-CoV S protein likely stimulates NF-κB via the upregulation of an upstream protein kinase C (PKC) isozyme PKCα, since S activated ERK and JNK were PKC dependent [192]. Although IL8 synthesis and secretion was not promoted by SARS-CoV E protein [204], deletion of E protein in recombinant SARS-CoV reduced NF-κB activation [203]. Additionally, overexpression of SARS-CoV N protein significantly increased NF-κB luciferase activity in a dose-dependent manner in Vero E6 cells, but not Vero, HeLa and Huh-7 cells, suggesting that this induction of NF-κB might be cell-specific [205,206]. On the other hand, co-immunoprecipitation experiments showed that SARS-CoV M protein physically bind to IKKb to sequester it in the cytoplasm, hence inhibiting the activation of NF-κB [207]. However, the MERS-CoV M protein did not affect the luciferase activity controlled by a promoter with NF-κB binding sites, although we cannot rule out the possibility that some other structural or non-structural MERS-CoV protein might be involved [133]. For HCoV-OC43, expression of its N protein alone was unable to activate NF-κB, unless under the stimulation of TNFα. This potentiation of NF-κB activation was through the interaction between HCoV-OC43 N and microRNA 9, which inhibits NF-κB [208].

#### 7.1.2. Nonstructural and Accessory Proteins

Previously, it was demonstrated that overexpression of SARS-CoV nsp1, but not HCoV-229E nsp1, could induce NF-κB activation [138]. The use of NF-κB inhibitor suppressed SARS-CoV nsp1-induced chemokine expression in a dose-dependent manner [209]. The PLpro domain found within nsp3 of SARS-CoV and MERS-CoV was shown to antagonize IFN and NF-κB activities [139,210]. However, in another study, SARS-CoV PLpro domain did not significantly negate the induction of expression of NFκB-dependent genes by Sendai virus infection [140]. Nevertheless, it was shown that SARS-CoV PLpro repressed NF-κB activation by removing K48-linked ubiquitination from IκBα [211].

Other accessory proteins of HCoVs have also been shown to interfere with NF-κB signalling. Expression of SARS-CoV 3a and 7a proteins was shown to significantly induce NF-κB-dependent luciferase activity. Enhancement of IL8 promoter activity by SARS-CoV 3a and 7a protein was negated by mutating the NF-κB binding site on the promoter [192]. Previously, expression of MERS-CoV ORF4a, but not ORF4b and ORF5, was shown to inhibit Sendai virus-induced firefly luciferase activity under the control of a NFκB-responsive promoter [133]. However, in another study, MERS-CoV ORF4b was demonstrated to moderately attenuate NF-κB-dependent luciferase activity induced upon TNFα treatment [212].

## 8. Conclusions

The relationship between a virus and its host is a complicated affair: a myriad of factors from the virus and the host are involved in viral infection and consequential pathogenesis. During viral infections, the host must respond to the virus by putting multiple lines of defence mechanisms in place. As intracellular obligate parasites, viruses have also evolved various strategies to hijack the host machineries. In this review, we first showed how viral factors could manipulate the host cell to expedite its own replication cycle and pathogenesis. We also highlighted how multiple cellular and viral factors come into play in their long-standing battle against one another. 

For years, HCoVs have been identified as mild respiratory pathogens that affect the human population. However, it was the emergence of SARS-CoV that thrust these human viruses into the spotlight of the research field. Therefore, most of the HCoV research today is pertained towards SARS-CoV. While the recent MERS-CoV outbreak has been mostly limited to the Middle East region, it is likely that more emerging or re-emerging HCoVs might surface to threaten the global public health, as seen from the high mortality rates in the past two outbreaks: SARS-CoV (10%) and MERS-CoV (35%). Therefore, study of the pathogenesis of all HCoVs would gain more insights for the development of antiviral therapeutics and vaccines.

## Figures and Tables

**Table 1:** Classification of human coronavirus.

Coronaviriniae Genera	Strains	Discovery	Cellular Receptor	Host	References
**Alpha-coronavirus**	HCoV-229E	1966	Human Aminopeptidase N (CD13)	Bats	[1,2,21]
	HCoV-NL63	2004	ACE2	Palm Civets, Bats	[3,21]
**Beta-coronavirus**	HCoV-OC43	1967	9-*O*-Acetylated sialic acid	Cattle	[4,5]
	HcoV-HKU1	2005	9-*O*-Acetylated sialic acid	Mice	[6,7]
	SARS-CoV	2003	ACE2	Palm Civets, Bats	[8,19,21]
	MERS-CoV	2012	DPP4	Bats, Camels	[9]
